# Evaluating the Protective Role of Vitamin A Supplementation in Periodontal Health: A Comprehensive Systematic Review and Meta-Analysis

**DOI:** 10.3390/jcm13164775

**Published:** 2024-08-14

**Authors:** Magda Mihaela Luca, Roxana Buzatu, Bogdan Andrei Bumbu

**Affiliations:** 1 Department of Pediatric Dentistry, Faculty of Dental Medicine, “Victor Babes” University of Medicine and Pharmacy Timisoara, Eftimie Murgu Square 2, 300041 Timisoara, Romania; luca.magda@umft.ro; 2 Department of Dental Aesthetics, Faculty of Dental Medicine, “Victor Babes” University of Medicine and Pharmacy Timisoara, Revolutiei Boulevard 9, 300041 Timisoara, Romania; 3 Department of Dental Medicine, Faculty of Medicine and Pharmacy, University of Oradea, 410073 Oradea, Romania; bogdanbumbu@uoradea.ro

**Keywords:** vitamin A, dentistry, periodontal disease

## Abstract

**Background**: Recent studies suggest a potential role for vitamin A supplementation in improving periodontal health, though evidence remains inconclusive. This systematic review and meta-analysis aimed to evaluate the protective role of vitamin A supplementation on periodontal health, focusing on outcomes such as gingival inflammation, pocket depth reduction, and alveolar bone preservation. **Methods**: A literature search was conducted in PubMed, Scopus, and Web of Science up until May 2024, adhering to strict inclusion criteria that required studies to involve human participants diagnosed with periodontal diseases and to assess the impact of vitamin A through dietary intake or supplementation. This review excluded studies not explicitly focused on vitamin A and those lacking clear, quantifiable outcomes. The risk of bias was assessed using the Newcastle–Ottawa Scale for observational studies and the Cochrane Collaboration’s tool for randomized controlled trials. Meta-analysis was performed to synthesize data and quantify the effectiveness of vitamin A on periodontal health outcomes. **Results**: A total of six studies were included in the final analysis with a total of 50,722 participants. The meta-analysis revealed a pooled odds ratio (OR) of 0.97 (95% CI: 0.94–1.00) for the association between vitamin A supplementation and periodontal health, indicating a slight protective effect. Notably, two high-quality studies reported ORs of 0.92 (95% CI: 0.85–1.00) and 0.83 (95% CI: 0.69–1.00), respectively, suggesting a potential reduction in periodontal disease risk with sufficient vitamin A levels. However, high heterogeneity (I^2^ = 86.93%) across studies indicates variability in outcomes, possibly influenced by demographic and lifestyle factors. **Conclusions**: Vitamin A supplementation may offer a marginal protective effect against periodontal disease, although results vary significantly across different populations and study designs. Further research is needed to clarify these relationships and to explore the mechanisms through which vitamin A influences periodontal health, considering the high degree of observed heterogeneity.

## 1. Introduction

Periodontal disease encompasses a variety of inflammatory conditions that affect the supporting structures of teeth, which can lead to severe dental and systemic health issues if left untreated [1,2]. The multifactorial etiology of periodontal diseases includes microbial dental plaque, genetic predisposition, and environmental factors such as smoking and nutrition. Among these, the role of nutrition has been increasingly recognized as crucial in both the prevention and progression of periodontal diseases [3,4,5].

Recent studies have highlighted the potential benefits of several micronutrients on periodontal health [6,7]. Vitamin A, known for its role in maintaining mucosal surfaces, has shown a protective effect in various diseases [8,9]. Similarly, other vitamins such as vitamin C, D, and E also exhibit protective roles. Vitamin C, crucial for collagen formation and gingival health, has been associated with improved oral health. Vitamin D is noted for its role in bone metabolism and has been linked with lower incidence of periodontitis through its effect on bone and tooth support [10,11]. Meanwhile, vitamin E, with its anti-inflammatory properties, contributes to the maintenance of periodontal health by modulating inflammatory responses [12,13].

The impact of dietary supplements on periodontal health has been explored, with evidence suggesting that the supplementation of these essential vitamins might reduce the progression of periodontal diseases, especially in populations at higher risk due to nutritional deficiencies. The role of supplements is particularly emphasized in settings where dietary intake might not be sufficient to meet the daily requirements [14,15,16,17].

Among the known contributors to oral health, there is vitamin C’s role in collagen formation and its importance in gum health, vitamin D’s contribution to calcium metabolism and its impact on teeth and jawbone health, and vitamin E’s antioxidant properties, which help to combat oxidative stress in oral tissues. Despite the recognition of micronutrients in periodontal health, there remains a significant gap in systematic evidence, particularly concerning the role of vitamin A. While some studies suggest its beneficial effects, the literature lacks a comprehensive meta-analysis that focuses solely on vitamin A’s impact on periodontal outcomes. This study aims to fill this gap by systematically reviewing and meta-analyzing the evidence to assess the protective role of vitamin A supplementation in periodontal health, addressing an outstanding research question about its specific contributions compared to other micronutrients. This approach is critical for delineating the unique benefits of vitamin A, potentially guiding future dietary recommendations and therapeutic interventions in periodontal disease management.

Given the promising but inconclusive evidence on the role of vitamin supplementation in enhancing periodontal health, this study aims to systematically review and meta-analyze the available literature to evaluate the protective role of vitamin A supplementation, specifically on periodontal health. The hypothesis of the study is that vitamin A supplementation will have a significantly protective role for periodontal disease outcomes compared to no supplementation or lower-serum vitamin A levels. The objectives include a thorough analysis of randomized controlled trials, observational studies, and clinical trials that have assessed the impact of vitamin A on various periodontal health parameters. This study seeks to provide a definitive answer to the potential benefits of vitamin A in the prevention and management of periodontal diseases.

## 2. Materials and Methods

### 2.1. Eligibility Criteria and Information Sources

For our systematic review and meta-analysis, we have delineated strict inclusion and exclusion criteria to ensure a focused and rigorous study selection process. The criteria set forth require that included studies must involve human participants who have been diagnosed with periodontal diseases. These studies must have specifically explored the impact of vitamin A on periodontal health, examining outcomes such as gingival inflammation, pocket depth reduction, and alveolar bone preservation through either dietary intake or supplementation. We also included diverse research designs such as randomized controlled trials, observational studies, cohort studies, case–control studies, and cross-sectional studies to ensure comprehensive coverage of the available evidence. The literature search was conducted in PubMed, Scopus, and Web of Science, including publications up until May 2024.

In terms of exclusion criteria, studies that do not focus explicitly on vitamin A, such as those assessing the effects of multivitamin supplements or general dietary patterns, were omitted. Additionally, studies that lack clear, quantifiable outcomes related to periodontal health or fail to provide sufficient detail for in-depth analysis were also excluded. To maintain the integrity and reliability of our review, the study excluded grey literature, including non-peer-reviewed articles, preprints, conference proceedings, and other non-peer-reviewed publications.

The following PICO statement was considered (Population, Intervention, Comparison, Outcome): In individuals diagnosed with periodontal diseases (Population), does vitamin A supplementation (Intervention) compared to no supplementation (Comparison) result in improved periodontal health outcomes such as reduced gingival inflammation, decreased pocket depth, and better alveolar bone preservation (Outcome)?

### 2.2. Search Strategy

Our search strategy incorporated the following keywords and Medical Subject Heading (MeSH) terms: “vitamin A”, “retinol”, “retinoic acid”, “carotenoids”, “beta-carotene”, “periodontal health”, “periodontitis”, “gingivitis”, “periodontal disease”, “dental health”, “gingival health”, “oral health”, “alveolar bone loss”, “dental plaque”, “immune response”, “inflammatory response”, “antioxidant effects”, “mucosal integrity”, “clinical outcomes” and “treatment efficacy”.

To structure the search effectively, we used Boolean operators to combine these terms in a manner that would refine and focus the search. The constructed search string was as follows: (((“vitamin A” OR “retinol” OR “retinoic acid” OR “carotenoids” OR “beta-carotene”) AND (“periodontal health” OR “periodontitis” OR “gingivitis” OR “periodontal disease” OR “dental health” OR “gingival health” OR “oral health”) AND (“immune response” OR “inflammatory response” OR “antioxidant effects” OR “mucosal integrity”) AND (“clinical outcomes” OR “treatment efficacy” OR “disease progression” OR “alveolar bone loss”))).

### 2.3. Data Collection and Selection Process

Following the Preferred Reporting Items for Systematic Reviews and Meta-Analyses (PRISMA) guidelines [18], our data collection and selection process were structured to ensure accuracy, reproducibility, and transparency. Initially, all retrieved records from the comprehensive database searches were independently screened by two reviewers. This step involved the assessment of each record based on the predefined inclusion and exclusion criteria to determine its eligibility for inclusion in our review.

Discrepancies between reviewers during this initial screening phase were addressed through discussion. If a consensus could not be reached, the issue was escalated to a third reviewer for a final decision. We used manual search and filter strategies to organize the records, remove duplicates, and ensure a smooth progression to the abstract and full-text review stages.

Each selected study was then subjected to a detailed evaluation where abstracts and full texts were reviewed to confirm their relevance and compliance with the inclusion criteria. The entire selection process was transparently documented and has been registered on the Open Science Framework (OSF), with all procedural details and the registration code made publicly available for verification and future reference osf.io/sczhm.

### 2.4. Data Items

The primary outcomes of interest were the clinical measures of periodontal disease, such as gingival inflammation, periodontal pocket depth, clinical attachment loss, and alveolar bone level changes. Additionally, we gathered information on participant demographics (age, gender, smoking status, and the presence of diabetes), study characteristics (country, year, design, and sample size) and the quality of studies to assess the representativeness and applicability of the findings. This systematic review defined periodontal health outcomes according to the standardized clinical parameters commonly used in dental research, such as the Community Periodontal Index (CPI) and bleeding on probing.

### 2.5. Risk of Bias and Quality Assessment

In assessing the risk of bias and the quality of studies included in our review, we adopted a dual approach tailored for both observational studies and randomized controlled trials (RCTs). For observational studies, we utilized the Newcastle–Ottawa Scale [19], a well-established tool that evaluates three core aspects: the selection of study groups, the comparability of the groups, and the determination of exposure or outcomes. Studies are awarded stars across these categories, culminating in an overall score that classifies the quality of each study as low, medium, or high.

For RCTs, we applied the Cochrane Collaboration’s tool for assessing the risk of bias [20], which examines several domains: random sequence generation, allocation concealment, the blinding of participants and personnel, the blinding of outcome assessment, incomplete outcome data, selective reporting, and potential for other biases. Each domain is assessed as having a ‘low risk’, ‘high risk’, or ‘unclear risk’ of bias. Two independent researchers conducted the assessments, with any discrepancies resolved through discussion or, if necessary, consultation with a third reviewer. This structured evaluation ensures that our conclusions are based on reliable and high-integrity data.

### 2.6. Synthesis Methods

Our synthesis methods combined both qualitative and quantitative approaches to evaluate the impact of vitamin A on periodontal health. We included studies that specifically provided data on vitamin A intake and its clinical effects on periodontal outcomes such as pocket depth, gingival inflammation, and alveolar bone preservation. Data were organized in tables, noting outcomes related to periodontal health improvements and levels of vitamin A intake. Missing data were identified, and their potential impacts on the findings were evaluated.

A meta-analysis was subsequently conducted to quantify the effectiveness of vitamin A supplementation or dietary intake on specific periodontal health outcomes. We assessed heterogeneity among the study results using the I^2^ statistic, which indicates the percentage of variation across studies that is due to heterogeneity rather than chance. High I^2^ values suggest significant variability, which is critical in interpreting the efficacy of vitamin A. All statistical analyses were carried out using standard software, with results presented alongside 95% confidence intervals to provide precise estimates of the effects observed.

## 3. Results

### 3.1. Study Selection and Study Characteristics

**Figure 1:**
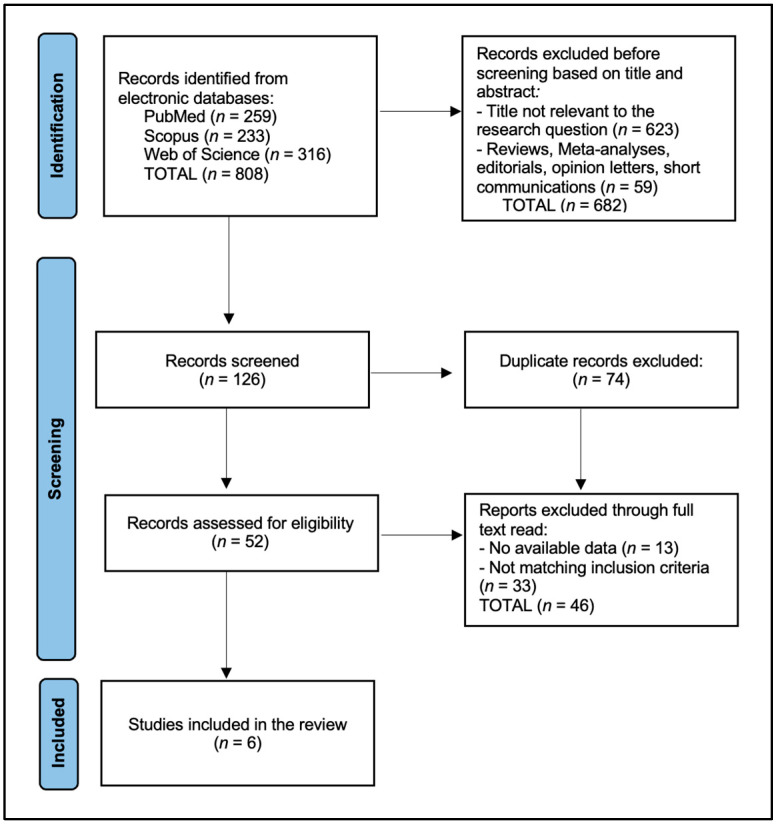
PRISMA flow diagram.

A total of 808 articles were identified according to the initial search, of which 74 duplicate entries were eliminated. From the remaining records, 682 were excluded before screening based on the title and abstract: 205 from PubMed (79% of initial PubMed findings), 225 from Scopus (97% of initial Scopus findings), and 252 from Web of Science (80% of initial WoS findings). After the full-text reviews, 46 articles were further excluded for not matching the inclusion criteria or having no available data; this comprised 10 from PubMed, 18 from Scopus, and 18 from WoS. The systematic review included a total of six eligible studies in the final analysis [21,22,23,24,25,26], as presented in Figure 1 and Table 1.

### 3.2. Results of Individual Studies

The total number of patients with periodontal disease across the studies amounted to 12,875, while those without the disease totaled 37,847. The analysis of the background characteristics of the patients enrolled in the systematic review, as presented in Table 2, revealed varied age demographics and gender distributions across six different studies that compared individuals with the different stages of periodontal disease to those without the disease. The studies included a wide range of participant numbers, from as few as 96 in the study by Linden et al. [26] to as many as 3994 in the study by Li et al. [22]. Age averages for patients ranged from 33.1 years in the youngest cohort (Park et al. [25]) to 64.2 years in the oldest (Linden et al. [26]), indicating a broad age range across the studies. Notably, the proportion of male participants varied significantly, from a low of 48.3% in the study by Zhou et al. [24] to 100% in Linden et al. [26], suggesting differing recruitment strategies or potential gender-related predispositions to periodontal disease severity (Table 2).

### 3.3. Results of Synthesis

The findings presented in Table 3 revealed a range of outcomes with regard to the impact of vitamin A levels on periodontal disease risk across different study populations. Chapple et al. reported a modest protective effect of higher vitamin A levels, with an odds ratio (OR) of 0.92 and a confidence interval (CI) ranging from 0.85 to 1.00, indicating that sufficient vitamin A could be associated with a decreased risk of periodontal disease development. Similarly, Li et al. found a protective effect with an OR of 0.83 (CI 0.69–1.00), suggesting that higher intake levels could potentially reduce the risk of periodontal damage. In contrast, Luo et al. reported an increased risk associated with insufficient vitamin A levels, with an OR of 1.78 (CI 1.53–2.39), emphasizing the negative impact of lower vitamin A levels on periodontal health.

Zhou et al. provided a more detailed analysis, noting significant variations in the effect of vitamin A across different subgroups, including age, racial background, smoking status, and other health conditions, with ORs ranging from 0.62 to 0.80, indicating a generally protective but variable effect. The other studies in the table showed a narrower range of effects, with Park et al. showing an OR of 1.15 (CI 0.81–1.62), which did not statistically signify protection or harm, and Linden et al. showing an almost neutral effect with an OR of 0.98 (CI 0.95–1.02). These variations highlight the complex interaction between vitamin A levels and periodontal health, suggesting that, while there may be a protective role, it is likely influenced by multiple factors, including systemic health conditions and lifestyle factors such as smoking and diabetes.

**Figure 2:**
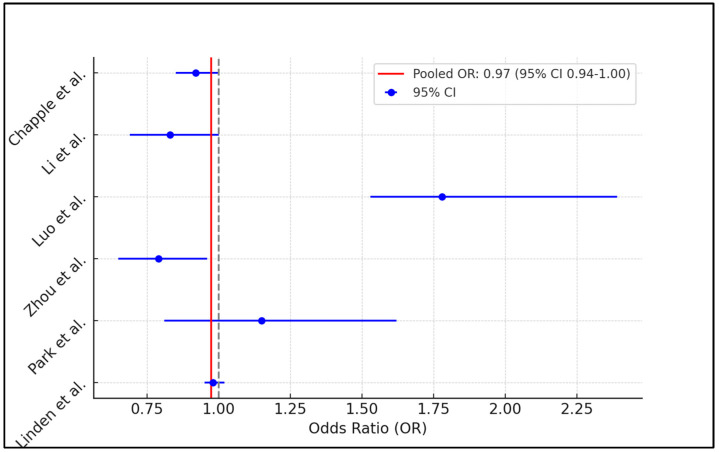
Forest plot of meta-analysis [21,22,23,24,25,26].

The meta-analysis of six studies investigating the effect of vitamin A on periodontal health resulted in a pooled odds ratio of approximately 0.97, with a 95% confidence interval ranging from 0.94 to 1.00. This suggests a marginally significant association between higher vitamin A intake and improved periodontal outcomes, indicating that vitamin A may confer a slight protective effect against periodontal disease. However, the high heterogeneity among the included studies, indicated by an I^2^ value of 86.93%, suggests considerable variability in how vitamin A affects periodontal health across different populations or under different study conditions, as presented in Figure 2.

## 4. Discussion

The findings from this systematic review and meta-analysis suggest a modest association between vitamin A supplementation and improved periodontal health outcomes, as evidenced by a pooled odds ratio of 0.97 (95% CI: 0.94–1.00). This result aligns with the hypothesized protective role of vitamin A, potentially attributable to its known effects on immune function and mucosal integrity. Notably, the studies by Chapple et al. [21] and Li et al. [22] demonstrated individual odds ratios that indicated a decreased risk of periodontal disease with higher vitamin A levels, emphasizing the potential for vitamin A to contribute to periodontal health. These findings underscore the importance of adequate vitamin A intake, especially in populations at risk of or currently experiencing periodontal disease.

However, the results exhibit significant heterogeneity, which may be influenced by several factors including variations in study design and the demographic characteristics and baseline nutritional status of participants. For instance, Zhou et al.’s study [24] highlighted that the impact of vitamin A varied significantly across different subgroups, suggesting that age, racial background, smoking status, and other comorbidities like diabetes, obesity, etc., may modify the effect of vitamin A on periodontal health. This variation is crucial for understanding the context-dependent nature of vitamin A’s protective effects and indicates that uniform supplementation recommendations may not be universally applicable. In evaluating the role of vitamin A in periodontal health, it is crucial to consider systemic factors like smoking and diabetes, which significantly influence outcomes. Smoking impairs immune responses and healing, potentially overshadowing vitamin A’s benefits, while uncontrolled diabetes exacerbates inflammation and attachment loss. Future research must adjust for these factors to accurately isolate vitamin A’s effects and ensure the findings reflect its true impact on periodontal health.

Furthermore, the studies included in this analysis employed varying methodologies to assess periodontal health and vitamin A levels, which might contribute to the observed heterogeneity. The clinical parameters used to define periodontal health, such as pocket depth and clinical attachment loss, were not standardized across studies, potentially leading to discrepancies in measuring the true effect of vitamin A on periodontal outcomes. Additionally, the assessment of vitamin A status through dietary questionnaires or blood serum levels introduces variability that could affect the accuracy and comparability of the results. In addressing the lack of standardization in the clinical parameters used to evaluate periodontal health, it is imperative to focus on a uniform metric that can reliably reflect the true impact of vitamin A on periodontal outcomes. Pocket depth and clinical attachment loss are both critical indicators, but for standardization, clinical attachment loss might be more suitable. This parameter measures the vertical distance from the cemento-enamel junction to the base of the periodontal pocket, providing a direct indicator of tissue destruction and periodontal support loss, which are crucial in assessing the progression and severity of periodontal disease. Standardizing clinical attachment loss as the primary metric could minimize variability across studies, enhancing the comparability and reliability of research findings concerning the influence of vitamin A on periodontal health.

In a similar manner, the study by Fawzy El-Sayed et al. [27] reviewed the role of various vitamins in periodontal wound healing and regeneration, highlighting the potential benefits of vitamins A, B, E, and CoQ10 in improving periodontal outcomes when used adjunctively with periodontal therapies. Their findings underline the heterogeneity and gaps in the current research, suggesting that, while some vitamins may have beneficial effects, the evidence remains inconsistent across different study models, including in vitro, animal, and clinical studies. Conversely, the study by Atalay et al. [28] focused specifically on the systemic use of retinoic acid, a derivative of vitamin A, and its effects on human β-defensin levels in saliva, serum, and gingival tissues. They found that systemic retinoic acid users had significantly reduced salivary hBD-2 levels compared to controls, with *p*-values of 0.042 and adjusted *p*-value of 0.031 for salivary hBD-2 concentrations, indicating a potential modulation of antimicrobial peptide expression by retinoic acid. However, no significant differences were noted in the serum or gingival tissue levels of hBD-1, hBD-2, or hBD-3, suggesting a localized effect of retinoic acid on salivary defenses rather than a systemic or tissue-specific response.

Moreover, the study by Yan Gao et al. [29] employed Mendelian randomization to explore the causal impact of diet-derived antioxidants on periodontitis, revealing that increased levels of circulating retinol significantly reduced the risk of periodontitis (pooled OR = 0.30, 95% CI: 0.15–0.61, *p* = 0.001), highlighting its protective effects. Conversely, Karim M Fawzy El-Sayed et al. [30]. demonstrated how ascorbic acid (AA)/retinol not only promoted the proliferation and stemness of gingival mesenchymal stem/progenitor cells but also enhanced their regenerative potential under inflammatory conditions, suggesting that these vitamins could play a crucial role in the regenerative therapies for periodontal diseases. Both studies underscore the significant, yet distinct, roles of specific vitamins in influencing periodontal health through different biological mechanisms and conditions.

In different parts of the world, the study by Patrícia Daniela Costa et al. [31] employed a cross-sectional multivariate analysis to explore the impact of micronutrient intake on periodontal status among adults utilizing a public health care system in Brazil. They identified that individuals in the poorest periodontal status cluster had lower intakes of key nutrients such as omega-3, fiber, zinc, potassium, copper, and vitamin C, with a prevalent periodontal pocket ≥4 mm in 67.4% of participants. Conversely, those with the healthiest periodontal status had higher educational levels and nutrient intake, demonstrating a clear link between micronutrient levels and periodontal health. Similarly, Karim M Fawzy El-Sayed’s study [32] investigated the influence of retinol and inflammatory cytokines on the stemness and differentiation potential of gingival mesenchymal stem/progenitor cells, finding that retinol significantly enhanced cellular proliferation and maintained pluripotency under inflammatory conditions.

In a similar manner, the study by Mayank Hans et al. [33] found that individuals with periodontitis exhibited significantly lower serum levels of certain vitamins compared to healthy volunteers, particularly noting statistically significant differences in β-cryptoxanthin, vitamin B12, and vitamin D levels (*p*-value < 0.05). These findings support the notion that deficiencies in specific micronutrients may contribute to the development of periodontal diseases. Correspondingly, the study by Muhammad H. A. Saleh et al. [34] explored the relationship between dietary supplements and periodontal health using a large dental Electronic Health Records database. They noted that, among various dietary supplements, only multivitamins and iron showed a significant positive association with periodontal health, whereas supplements like folic acid and vitamin E were associated with a higher risk of periodontitis.

This systematic review underscores the potential of vitamin A supplementation to enhance periodontal health by reducing gingival inflammation and preserving alveolar bone integrity. These results are clinically significant, suggesting that vitamin A could be a valuable adjunct in the treatment protocols for periodontal diseases, especially in patients with known nutritional deficits. Future research should aim to delineate the specific biological mechanisms through which vitamin A impacts periodontal tissues and to verify these effects in diverse patient populations through well-designed, randomized controlled trials with robust outcome measures. This could lead to more personalized dietary recommendations and supplementation strategies in stomatology.

This study is not without limitations. The high heterogeneity observed among the included studies poses a significant challenge, possibly diluting the overall conclusions that can be drawn regarding the efficacy of vitamin A supplementation. Future research should aim to standardize the assessment methods for both periodontal health and vitamin A status to improve comparability across studies. Moreover, this review included only cross-sectional studies; therefore, longitudinal studies are needed to establish the causality and the long-term effects of vitamin A supplementation on periodontal health.

## 5. Conclusions

In conclusion, the evidence from this systematic review and meta-analysis supports a slight protective effect of vitamin A supplementation on periodontal health. Despite the variability in findings, which may be influenced by demographic and lifestyle factors, vitamin A appears to play a beneficial role in periodontal disease management. These findings highlight the potential for dietary interventions to enhance periodontal health, particularly through targeted vitamin A supplementation in at-risk populations. As such, incorporating vitamin A-rich foods or supplements could be considered as part of comprehensive strategies for periodontal disease prevention and management, although tailored approaches based on individual risk profiles and dietary habits may be necessary to optimize outcomes.

## Figures and Tables

**Table 1:** Characteristics of the studies included in the review.

Study and Author	Country	Study Year	Study Design	Quality Assessment
1 [21] Chapple et al.	USA	2007	Cross-sectional	Medium
2 [22] Li et al.	China	2022	Cross-sectional	Medium
3 [23] Luo et al.	China	2018	Cross-sectional	Medium
4 [24] Zhou et al.	USA	2023	Cross-sectional	High
5 [25] Park et al.	South Korea	2016	Cross-sectional	Medium
6 [26] Linden et al.	UK	2009	Cross-sectional	High

**Table 2:** Patients’ background characteristics.

Study and Author	Number of Participants (Study Groups)	Comparison Group	Age (Category/Mean/Median), Years	Gender (Male)
1 [21] Chapple et al.	Mild disease: 1567 patients Severe disease: 609 patients	No periodontal disease: 20,784	Mild disease: 52.2 Severe disease: 56.4	Mild disease: 61.1% Severe disease: 68.1%
2 [22] Li et al.	Moderate/severe periodontitis: 3994 patients	No periodontal disease: 4965	56.7	57.9%
3 [23] Luo et al.	Moderate disease: 2274 patients Severe disease: 676 patients	No periodontal disease: 3465	Moderate disease: 55.3 Severe disease: 54.5	Moderate disease: 54.1% Severe disease: 70.4%
4 [24] Zhou et al.	Moderate/severe periodontitis: 3380	No periodontal disease: 5701	50.8	48.3%
5 [25] Park et al.	Periodontitis: 279 patients	No periodontal disease: 1770	33.1	52.3%
6 [26] Linden et al.	Periodontitis: 96 patients	Mild/no periodontal disease: 1162	64.2	100%

**Table 3:** Evaluation of the risk of periodontal disease development.

Study and Author	Periodontal Disease Assessment	Smoking Status and Diabetes Status	Vitamin A Assessment	Risk Assessment (OR/HR/RR—95% CI)
1 [21] Chapple et al.	At least one site with both clinical attachment loss ≥4 mm and probing pocket depth of ≥4 mm	Mild disease (smoking): 25.8% Severe disease (smoking): 21.4% Mild disease (diabetes): 12.2% Severe disease (diabetes): 15.3%	1st quintile: 1.33 umol/L 2nd quintile: 1.68 umol/L 3rd quintile: 1.92 umol/L 4th quintile: 2.20 umol/L 5th quintile: 2.69 umol/L	Sufficient vitamin A OR: 0.92 (95% CI: 0.85–1.00)
2 [22] Li et al.	≥2 Interproximal sites with a clinical attachment loss (CAL) of ≥4 mm; ≥2 Interproximal sites with a periodontal probing depth of ≥5 mm	Smoking: 17.8% Diabetes: 21.2%	Protective daily vitamin A cutoff: 526.7 retinol activity equivalents	Sufficient vitamin A OR: 0.83 (95% CI: 0.69–1.00)
3 [23] Luo et al.	At least two interproximal sites with PD of at least 5 mm not occurring on the same tooth, or at least two interproximal sites that are not on the same tooth and that have an AL of at least 4 mm	Moderate disease (smoking): 29.8% Severe disease (smoking): 27.4% Moderate disease (diabetes): 15.4% Severe disease (diabetes): 13.6%	Moderate disease ≤261 ug: 26.5% Severe disease ≤261 ug: 31.0%	≤61 ug (insufficient) vs. ≥786 ug (sufficient) OR: 1.78 (95% CI: 1.53–2.39)
4 [24] Zhou et al.	Moderate periodontitis: ≥2 interproximal sites with PD ≥5 mm not on the same tooth, or ≥2 interproximal sites with CAL ≥4 mm not on the same tooth; Severe periodontitis: ≥2 interproximal sites with CAL ≥6 mm not on the same tooth and ≥1 interproximal site with PD ≥5 mm	Smoking: 16.7% Diabetes: 9.5%	NR	(ORtertile3vs1 = 0.79, 95% CI: 0.65–0.96). The association was still significant in populations who were less than 60 years old (ORtertile3vs1 = 0.80, 95% CI: 0.65–0.97), non-Hispanic black (ORtertile3vs1 = 0.62, 95% CI: 0.42–0.94), PI ≤ 1.3 (ORtertile3vs1 = 0.72, 95% CI: 0.55–0.93), 1.3 < PI ≤ 3.5 (ORtertile3vs1 = 0.70, 95% CI: 0.55–0.89), non-smokers (ORtertile3vs1 = 0.63, 95% CI: 0.48–0.81), obesity (ORtertile3vs1 = 0.68, 95% CI: 0.49–0.94), and not exhibiting diabetes mellitus (ORtertile3vs1 = 0.79, 95% CI: 0.65–0.95) or hypertension (ORtertile3vs1 = 0.63, 95% CI: 0.47–0.84).
5 [25] Park et al.	Periodontitis was defined as a CPI greater than or equal to 3, which indicates that at least one site had a probing pocket depth of >3.5 mm (code 4 > 5.5 mm)	Smoking: 18.7% Diabetes: 17.1%	Periodontitis <602 ug: 50%	Insufficient vitamin A OR: 1.15 (95% CI: 0.81–1.62)
6 [26] Linden et al.	Severe periodontitis >15% of all sites measured had a loss of attachment (6 mm), and there was at least one site with deep pocketing (6 mm)	Smoking: 36.4% Diabetes: 11.5%	NR	OR: 0.98 (95% CI: 0.95–1.02)

NR—not reported; OR—odds ratio; RR—risk ratio; PD—periodontal disease; CPI—Community Periodontal Index; PD—pocket depth; CAL—clinical attachment loss.

## Data Availability

Not applicable.
